# Ebola virus disease outbreak in Liberia: application of lessons learnt to disease surveillance and control

**DOI:** 10.11604/pamj.supp.2019.33.2.19074

**Published:** 2019-05-24

**Authors:** Ibrahima Socé Fall

**Affiliations:** 1 World Health Organization, Regional Office for Africa, Brazzaville, Congo

**Keywords:** Ebola, Outbreak, Disease surveillance and control, Liberia

## Editorial

The outbreak of Ebola virus infection in West Africa countries, including Liberia, was the largest on record in terms of the unprecedented number of reported cases (n= 28,616), out of which 15,227 were confirmed and 11,310 deaths in Guinea, Liberia, and Sierra Leone and its’ rapid transmission in dense urban populations [1]. An additional 36 cases and 15 deaths occurred in Italy, Mali, Nigeria, Senegal, Spain, United Kingdoms and United States [2]. With an estimated mortality rate at around 70%, including massive death tolls in health care workers, the epidemic undermined the hitherto fragile health-care systems and presented public health challenges that have never been encountered previously and which were further constrained with the absence of treatment and vaccination options.

Liberia was most hit by the outbreak given its fragile health system. Before the outbreak of the Ebola epidemic, Liberia struggled with a very weak health system that was devastated and weakened by a protracted civil war. It had just 50 doctors for its 4.3 million population with poor capacity to respond to an epidemic of that magnitude [3]. The Ministry of Health, with support from WHO, led the response to stop the outbreak and together with partners, mobilized both human and material resources to end the outbreak in 2015.

By the end of the outbreak a total of 4810 deaths were recorded out of 10678 confirmed cases in Liberia [2]. The epidemic had severe and devastating impacts on the health system of Liberia, including health workforce and supply chain. It stalled progress across the health sector, including progress towards achieving the MDGs. It caused a deceleration of progress in reducing mortality due in part to the deaths resulting from Ebola directly. Similar deceleration in the mortality rate due to malaria could be attributed to the disruption to malaria treatment interventions [4]. Similarly, in 2014, DTP3 coverage dropped in Liberia from 76% in 2013 to 50% in 2014 [5]. A 50% reduction in access to healthcare services during the Ebola outbreak was estimated and this exacerbated malaria, HIV/AIDS and tuberculosis mortality rates [5]. Other services affected included limited access to Caesarian sections and clinic attendance of under-5 children, with a likelihood of a resultant high morbidity and mortality.

However, a number of lessons were learnt in the process of responding to the outbreak. Some of these lessons have been deployed to strengthening the health system in Liberia and preparing it to respond not only to Ebola outbreak but the outbreak of any epidemic prone diseases. These lessons have actually been successfully applied to enhanced disease surveillance and response in Liberia. Other important areas where they have been applied include response to meningococcal disease cluster in Foya district, Lofa County, Liberia January to February 2018; risk Communication during disease outbreak response in post-Ebola Liberia: experiences in Sinoe and Grand Kru Counties; as well as detecting and responding to recurrent measles outbreak in Liberia post Ebola-Epidemic 2016-2017 among others.

This special issue of the Pan African Medical Journal documents the successful application of lesson learnt from response to the Ebola virus disease outbreak in Liberia and identifies some best practices that could be applied to other disease prevention, control and elimination programmes with similar success. The papers, written by those who were actively engaged in response to epidemics in Liberia, cover critical topics in from preparedness and response to disease outbreaks, strengthening surveillance systems and integration as well as strengthening health workforce post Ebola virus disease outbreak. The comprehensive analysis of the success stories made in disease control and epidemic response in Liberia with clear discussion of accompanying challenges presented in this special edition is extremely useful and timely. The articles carefully chart the successes in turning tragedy to gains!

## Competing interests

All the authors declare no competing interest.
